# Promoting youth engagement in agriculture through land titling programs: Evidence from Tanzania

**DOI:** 10.1016/j.heliyon.2024.e29074

**Published:** 2024-04-04

**Authors:** Haji Athumani Msangi, Betty Waized, Daniel Wilson Ndyetabula, Victor M. Manyong

**Affiliations:** a Rwanda Institute for Conservation Agriculture, Gashora, Bugesera, Rwanda; b Department of Agricultural Economics and Agribusiness, Sokoine University of Agriculture, P.O. Box 67104, Morogoro, Tanzania; c International Institute for Tropical Agriculture (IITA), P.O. Box 34444, Dar es salaam, Tanzania

**Keywords:** Land titling, Youth, Participation in agriculture, IPWRA, Land tenure security, Tanzania

## Abstract

In many African countries, land access and tenure insecurity pose significant challenges to agriculture, in particular for the youth. As the farming population ages, young people are expected to take over, but they don't often show much interest in farming, which could harm the future of agriculture in Africa, where the population is the youngest. Land reforms and titling programs are suggested as amongst strategies to make agriculture more attractive to investors and promote youth involvement. As a result, majority African countries undertook reforms such as land titling, ownership mapping and market facilitation as policy prescriptions for promoting youth involvement in agriculture. Nonetheless, the impact of these programs is not well documented in the body of literature thus constraining policy decisions. This study examines the impact of land titling on youth participation in agriculture in Tanzania, using 2020/2021's wave of Tanzania national panel survey data with a sample size of 2725 youth households from 419 enumeration areas. The study finds that land titling is a critical factor in promoting youth participation in agriculture in Tanzania, with young people who have titled land tending to allocate more resources (time) to farming activities. The study also identified farm size, educational level, and land dispute experience as significant factors influencing youth participation in agriculture. Based on these findings, the study recommends interventions to promote youth participation in agriculture in Tanzania, including investing in land titling programs that specifically target young people and promoting educational programs that equip young people with foundational skills. The study also highlights the need for tailored interventions that address the specific needs of different groups of young people. Overall, the study underlines the importance of promoting youth participation in agriculture in Tanzania and by extension to other African countries to contribute to food security and rural development.

## 1 Introduction

### 1.1 Background information

Land tenure and land right issues in Africa have been in the midst of the critical matters in development agendas of many African countries. This is due to the fact that rural poverty in these countries is largely connected with lack of access to land and tenure insecurity [1]. It is especially critical in countries like Tanzania and others in Sub-Saharan Africa (SSA) where agriculture is the primary economic activity and land tenure security is important for ensuring household food needs and income generation [2,3]. The literature indicates that the farming population is aging globally where the average age of farmers is 60 years old [4]. Despite the observed trend of aging farming population, young people are increasingly involving in non-farming careers rather than succeeding the footpaths of their parents and grandparents [4]. The future agricultural sector, especially in most African countries, may be glooming if farming is entirely left in the hands of aged subsistence farmers who currently dominate the farming population. This is because the productivity of this aged group farmers may not be enough to meet the food needs of the rapidly growing population as well as meeting the needs of the industries in raw materials. Also, these farmers are likely to leave farming on account of age [4].

Youth population, as defined in Tanzania as people aged between 15 and 35 years old, presents a great prospect as the entrepreneurial and innovative energy and capacities of this group can help rejuvenate the agricultural sector and enhance growth of the local economies [5] This is especially relevant in Tanzania and in other African context where the population is the youngest in the world and about 60–70 percent of the population is below 30 years of age [6].

However, youths do not mechanically venture into agriculture, rather for them to see agriculture as a lucrative and thrilling venture and career path, youth need access productive resources such as land and financial capital in complement with entrepreneurial education and respective technical training (ibid). In the context of Tanzania, majority of youths live in rural spaces where the livelihoods are mainly derived from agriculture [7]. Nevertheless, the problems of rapid population growth associated with rapid urbanization and the rising interest of foreign agricultural investors are increasing pressure on agricultural land, thus, resulting into a serious problem of localized land scarcity in Tanzania [8]. In most Sub-Saharan African (SSA) countries where land markets are highly inefficient, credit markets are very thin and where there are few or no large farm investments that would offer enough farm wage employment opportunities to the rural households, access to secure agricultural land is a key determinant of the fate of most youths with regards to their livelihood strategies and choices [7]. That is, whether youth would choose to derive their livelihoods from agriculture or otherwise migrate to urban areas in search for non-farm wage employment opportunities. Many studies indicate equitable access to productive land and tenure security as a major source of financial security through the use of land as collateral to secure credit from financial institutions or transfer of ownership through land selling, renting, sharecropping and/or donate [9,10,11,7]. Furthermore, land tenure security creates motivation for investment and conservation, thus, sustaining and enhancing the value of land and its productive capacity [12]. However, a plethora of studies indicate that limited land access, unequitable distribution and transferability, and disputes are among serious challenges surrounding the land administration systems of most SSA countries [13,9,14]. In that regards, land tenure and property right systems in Tanzania are highly insecure, thus, hampering sustainable agricultural investments, land management efforts, credit access and proper functioning of the land markets [15,10,12,16]. The above issues facing the Tanzania's administration systems along with other problems that agricultural sector is encountering make farming lesser or even not attractive livelihood strategy for most rural youth thus hampering their active participation in agribusiness, given the fact that youths are among the most vulnerable groups to the aforementioned problems.

In response to the issues of poor land administration, land reforms and titling programmes have widely prescribed as a solution to make agriculture an attractive venture for domestic and international investors thus promoting youth participation in agribusiness [17,6,18].

### 1.2 Land tenure systems in Tanzania

Tanzania has pluralistic land tenure approach where both customary, statutory systems as well as mixed systems are recognized [19]. The tenure pluralism existed since the colonial era where there was ownership structure that encompassed common and decree law in access, control and use of land under this ownership structure colonial leaders consigned customary law to the indigenous community in majority of African countries [20,21]. Tanzanian land tenure system is dominated by customary which accounts for more than 70% of the total owned land and only 2 percent of the landowners have legal ownership documents [22].

In the 1990s, Tanzania undertook comprehensive reforms of land laws and policies to transform the dominant informal systems of land tenure into more standardized formal systems of tenure to promote equitable and secure land access and ownership for rural and urban households for inclusive socio-economic development [23]. Furthermore, as an attempt to turning agricultural land from seemingly dead asset into more valuable and productive asset, the Government of Tanzania in partnership with Hernando de Soto's Peruvian Institute of Liberal Democracy (ILD) and Norwegian Government established a Property and Business Formalization Programme, in 2003, which is best known in Kiswahili language acronym as MKURABITA [24]. The programme aimed at formalizing land ownership in the country, where about 89 percent of properties are “extra-legal”, through systematic registration and titling of land for efficient and effective land administration in Tanzania [24]. The programme definition of “extra-legal” properties is that the properties under “extra-legal” ownership can neither be easily traded nor used as security to secure credit from the banks [24]. Several other land titling programs have been implemented in Tanzania which include the “granted rights of occupancy” for general land, “customary rights” for village land, mortgage of Certificate of Customary Right of Occupancy (CCRO), Oxfam's land titling initiative, the land management program and several others [20,24,22,25]. The expected outcomes of titling are, among others, to revamp the land markets in Tanzania, promote access to credit, ensure property security, lower transaction costs for property right exchange.

Many developing countries and especially African countries have implemented land tenure formalization and certification programs to promote socio-economic development and poverty reduction. However, many empirical studies have failed to generate conclusive empirical evidence supporting the importance of land titling interventions to socio-economic development. Unlike Asia's and Latin America's context where most empirical evaluations (for example, Galiani and Schargrodsky [26] and Holden, Deininger and Ghebru [27]) of the impact of titling programs have confirmed the impact of these programs, empirical evidence in Africa remains mixed and conflicting. While some studies found positive impact of land titling interventions on key outcomes such as land tenure security, investments, credit access and productivity [9,10,12,27,28], others such as Place and Migot-Adholla [1], Ghebru and Holden [12], Holden, Otsuka and Place [11] found no evidence to confirm the effect on some of the key outcomes.

In addition, most empirical assessments of the impact of titling interventions in this domain encounters serious methodological challenges related of selectivity biases and endogeneity problems given the sporadic and non-compulsory nature of many land registration and certification programs.

This study is carried out to examine the effect of land titling programs that have been implemented in Tanzania on youth's decision to participate in agriculture and its implication on youth farm productivity while addressing the methodological challenges of previous studies.

Following Section 1 on the introduction, Section 2 describes the study methodology; Section 3 presents the results and their discussions, and Section 4 concludes and offers policy recommendations and suggests areas for further research.

## 2 Methodology

### 2.1 Conceptual framework

**Fig. 1:**
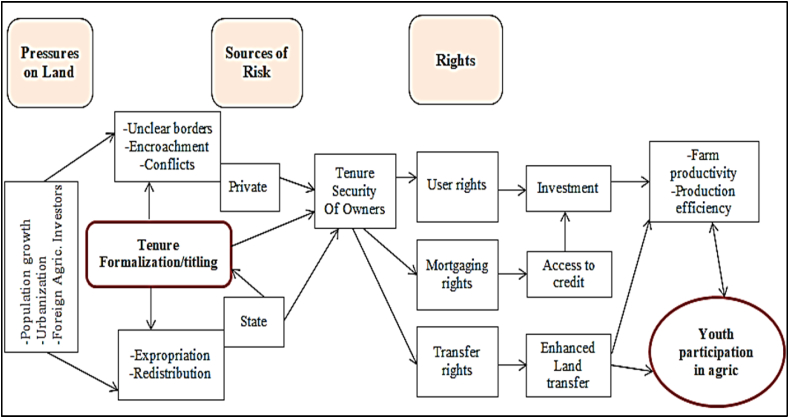
Conceptual framework (Source: Adapted and modified from Holden and Ghebru [16] and Platteau [34]).

The conceptual framework of this study is based on economic rationality of the youth's decisions as an economic agent in choosing and allocating scarce economic resources on land or somewhere else (non-farm sector). It designates the way in which land tenure security could affect youth's decision to invest in land (farming) or non-farming as one of livelihood options with a certain probability of reaping possible returns from their investment in given period of time. In countries like Tanzania where non-agricultural sectors are not well developed to offer lucrative investment opportunities and wage employment, access to land is an important determinant of whether youths in the rural agrarian economies would engage themselves in agriculture [29]. According to Holden, Otsuka and Place [11], the motivation to invest in land is significantly affected by the expected time period given which the returns on investments are realized. This is mainly because the expected returns on investment are greatly dependent on the level of perceived risks such as expropriation by the Government or powerful groups or encroachment by the other people or unanticipated changes in the overall economy sector, etc. The fact that land administration systems in Tanzania are considered inefficient, land transactions related to sale or purchase, rental/lease, inheritance, donating to others and sharecropping), youth land access and tenure security are highly hampered. Along with poor land administration systems in Tanzania and other Sub-Saharan African countries, is a silent but serious issue of rapidly growing pressure on agricultural land induced by rapid population growth, urbanization, climate change, and upsurge of foreign agricultural investors [30,31,32]. All these challenges increase the perceived risk and reduce the willingness of investing in agricultural land which in turn make agriculture less attractive to youth. Land formalization programs would be expected to increase the youths' perceived tenure security as argued in the coasian theorem thus affecting both supply and demand of land markets as it would reduce the perceived investment risk. This increases incentives to both landowners and renters [33,12]. Increased land tenure security is likely to increase youths' confidence to invest in or rent-out the formalized land. This is because titling induced improved tenure security would increase the probability that farmers would realize the expected returns from the land investments. In addition, well implemented land formalization could increase the collateral value of the titled which would thus improve farmers' access to credit to finance agricultural investments which would in turn foster inclusive agricultural and rural development and economic development at large. Such a secured tenure of that leads to enhanced farm productivity and increased production efficiency will stimulate youth participation in agriculture. The above relationships are depicted in Fig. 1. This conceptual framework of this study is the guide to data sources, sampling size and data analysis.

### 2.2 Theoretical framework

This study is underpinned by theory of the firm and property rights theory. The standard producer theory establishes that producers are rational economic agents who strive to maximize their profit which entails utility maximization [35]. Economic agents strive to optimize this objective subject to a number of constraints. The nature and type of property rights in the economy affect producers’ incentive and hence the decision to allocate their scarce resources in production activities [36]. With regards to the land titling, this study largely borrows from the property rights theory according to which the nature of property rights in the economy has profound implications on resources allocation efficiency [37]. In the context of land as a property, the way land rights are defined, and the nature of land tenure systems affects the level of investment activities in land as well as initiatives pursued by landowners and users to protect and sustain their land. Therefore, the core role of property rights in any economy is to provide for producers to efficiently allocate their scarce resources in a manner that is sustainable and internalizes possible externalities [38,39]. Additionally, this theory contends that property rights interventions arise to solve the economic problem scarce resource allocation that would promote security and confidence on both demand and supply side of the markets. This would result into proper functioning of facto markets, particularly land markets thus providing incentives to economic agents to act more rationally in resource allocation [34]. According to Coase [37] well defined property rights act as incentives for investment that would allow efficient resource allocation and hence economic growth and development.

### 2.3 Data source

This study uses the Living Standard Measurement Studies-Integrated Surveys in Agriculture (LSMS-ISA) data for Tanzania National Panel Survey (NPS) data disaggregated into Tanzania mainland and Zanzibar. These are nationally representative household survey data which provide for various measures of poverty, agricultural production as well as several other key socio-economic development indicators from 2008/09 to 2020/21. The LSMS-ISA NPS covers a wide range of socio-economic aspects including but not limited to education, gender, health, income and others. As previously stated, there are several panel data available from 2008/09 to 2020/21, but as far as the study objectives are concerned, this study uses only last panel cross-section (2020/21) which among other things the named wave (2020/21) contains information on land ownership documentation, tenure systems and tenure security. Previous waves are equally important as the last wave, but the only challenge is that they don't contain sufficient information to answer the study objectives. Another advantage of using this wave is that there is an added value in using the most recent data in analyzing these cross-cutting land tenure issues.

### 2.4 Sampling

The study conducts its analyses at the plots and household levels. At plot level, the analyses include farming plots that households own with either formal documentations or just informally. At the household level, the analyses include households with at least single agricultural plot of land. The sample size of 3555 agricultural plots from 2725 youth households from 419 enumeration areas is used in this study.

### 2.5 Econometric analysis

Land titling systems in most SSA countries, including Tanzania, are more of sporadic than systematic and non-compulsory nature where landowners can apply for land certificates on their own initiatives [15,2,22]. Given this nature of land titling systems in Tanzania, it can, therefore, be affirmed that the youth households that own land certificates are, in essence, not the random sample of the entire household population in Tanzania. This indicates a potential for selectivity problem in titling impact evaluations that lead into biased estimates of the impact. To account for the potential selection bias, this study uses Cattaneo's [40] doubly robust Inverse-Probability Weighted Regression Adjustment (IPWRA) approach to tackle the estimation issues. The aim is to model the effect of land titling on youth participation in agriculture in Tanzania. The IPWRA approach consists of two stages: in the first stage, treatment status (land titling) is predicted, and in the second stage, outcome (youth participation in agriculture) is estimated through weighted regression. IPWRA is doubly robust because it produces unbiased estimates of the treatment effect even if only one of the treatment or outcome models is correctly specified. This property is supported by Abadie and Imbens [41], Abadie and Imbens [42], Imbens [43], Imbens and Wooldridge [44], and Wooldridge [45]. IPWRA also allows for multiple treatments, which is relevant in this study.

While endogeneity problem is widely cited in the assessment of land titling program impacts, owing to the non-sporadic nature of these programs, it has been often overlooked in the literature, potentially resulting in misleading conclusions [40,46]. The IPWRA method effectively addresses this issue by incorporating inverse-probability weights, facilitating a more precise estimation of the impact of land titling on youth participation in agriculture. This not only significantly enhances the robustness of the study's findings but also aligns with the double robustness property of the IPWRA method, ensuring unbiased estimates even if either the model predicting land titling or the model predicting youth participation is mis-specified. This dual robustness further fortifies the reliability of the study's conclusions, advancing our understanding of the impact of land titling on youth engagement in agriculture in the specific context of Tanzania.

Despite the above strengths of IPRWA method, its effectiveness in controlling for unobservable heterogeneity is limited (Abadie and Imbens, 2006). The method relies heavily on observable characteristics included in the models, and if relevant unobservable factors are omitted, the potential for bias persists. In the context of this study, factors such as social networks and individual motivations that are unlikely to be explicitly captured may potentially introduce bias into the estimated impact of land titling on youth engagement in agriculture. This limitation is particularly relevant in the case of land titling programs in Tanzania, where the sporadic and non-compulsory nature of the systems introduces complexities that may not be explicitly captured by the covariates. To minimize the potential unobservable heterogeneity bias, the study includes as many covariates as possible in the models as well as relevant covariates such as group memberships (cooperative and SACCOS membership) as proxies for social networks, and possession of dwelling certificate and/or other land tenure documents, and formal education levels as proxies for individual motivations for participation in land titling [9]. However, the challenge remains in identifying and measuring all relevant factors comprehensively. The difficulty in eliminating this potential source of bias emphasizes the importance of interpreting the study's findings with caution and recognizing the inherent limitations of the employed estimation strategy.

#### 2.5.1 Binary logit selection model

A binary logistic model is be employed to predict the propensity scores and estimate the socio-economic determinants of youth land titling decisions by regressing land titling status on set of observable socio-economic characteristics. The model is specified as follows:

(1) $$P\left(T_{i}=1|Z_{i}\right)=\frac{e^{Z_{i}}}{1+e^{Z_{i}}}$$

where $P_{i}$ represent the probability that *i*th youth household has a land certificate (i.e *T* = 1), e is base of natural log and Z is a function of a set of observable characteristics that determine probability that an *i*th youth household has a land certificate. Z is further defined as follows;

(2) $$Z_{i}=\beta_{0}+\sum_{i=1}^{n}\beta_{i}X_{i}+\varepsilon_{i}$$

By incorporating the above Eq. (2) into Eq. (1), a new version of Eq. (1) can be obtained as shown below in Eq. (3):

(3) $$P\left(T_{i}=1|X_{i}\right)=\frac{e^{\left(\beta_{0}+\sum_{i=1}^{n}\beta_{i}X_{i}+\varepsilon_{i}\right)}}{1+e^{\left(\beta_{0}+\sum_{i=1}^{n}\beta_{i}X_{i}+\varepsilon_{i}\right)}}$$

where, X represents a set of observable socio-economic characteristics that determine the probability that certain youth household will have a land certificate. $\beta_{0}$, $\beta_{i}$ are the regression coefficients to be estimated and $\varepsilon_{i}$ is an error term which is assumed to follow a logistic distribution [47,48].

The above logit model is estimated by maximum likelihood estimation technique from which the coefficients and propensity scores is obtained [48,49].

#### 2.5.2 Regression Adjustment estimator

In the second stage, separate ordinary least square regressions are run based on Manda et al. [50]'s specification and the Regression Adjustment (RA) estimator is used to predict treatment specific outcomes for each participant. The outcome model as specified in Eq. (4) is estimated using the same set of covariates ($X_{i}$) used in the treatment model:

(4) $$Y_{i}=X_{i}\beta_{k}+\mu_{i}$$

where $Y_{i}$ is potential outcome of the treatment *k*, $\beta_{k}$ represents the coefficients of the outcome model and $\mu_{i}$ is the error term. The $\beta_{k}$ is estimated as shown in Eq. (5) below:

(5) $$\beta_{k}=\min\sum_{i=1}^{N}\frac{{\left[Y_{i}-Z_{i}\beta_{k}\right]}^{2}}{\mathrm{Pr}\;\left(T_{i}=k\right)}$$

where *N* represents the sample size.

The average treatment effect (ATT) of receiving treatment *k* relative to alternative treatments, *j*, is computed by averaging the predicted potential outcomes of different treatment categories and its estimator is expressed in Eq. (6) as follows:

(6) $$ATT=E\left[Y_{i1}-Y_{i0}\right]=\frac{1}{N_{1}}\sum_{i=1}^{N_{1}}\left[\frac{Y_{i1}}{\mathrm{Pr}\;\left(T_{i}=1\right)}-\frac{Y_{i0}}{\mathrm{Pr}\;\left(T_{i}=0\right)}\right]$$

where; $N_{1}$ is the number of youth farmers whose land is titled

As pointed out earlier that unlike other non-experimental impact estimators, the IPWRA uses weighted regression approach to estimate the average treatment effect on the treated to obtain the IPW of the treatment as described in Cattaneo [40]. This makes IPWRA consistent even when either of the treatment or outcome equations is not correctly specified, but not both, giving its doubly robust nature.

The description of explanatory and independent variables used in the empirical model for this study are described in Table 1.

**Table 1:** Description of explanatory and independent variables.

Dependent Variables	Name/Description	Measurement and further description
	Youth Participation in Agriculture	1 if a youth household owns a farm or participate in off-farm agric-activities, 0 Otherwise
	Land titling	1 if a youth household own a formal land certificate, 0 Otherwise
Explanatory Variables	**Household characteristics**	
	Sex of household head	1 for Male, 0 for Female
	Education	Number of years of schooling of the household head
	Family Size	Number of people in each household
	Age	Age of household head (number of years of life)
	Extension services	Number of contacts with extension agent per year
	Farming experience	Number of years of in farming
	Social capital	1 if a youth household has a close connection with political or high-profile leaders in the village, 0 otherwise
	Number of plots owned	Number of plots owned by the youth household
	**Plot characteristics**	
	Holding size/cultivated area	Hectares
	Years of holding	Number of years since acquisition
	Distance from home to plot	Kilometers
	Distance from plot to market	Kilometers
	Distance from plot to market	Kilometers

## 3 Results and discussion

### 3.1 Descriptive results

The descriptive results presented in Table 2 show that youth spend an average time of 17.759 h per week in the farming. Specifically, youth with formal land titles spend significantly more time (21.728 h per week) in the farm, equivalent to 24% more hours per week on farming, compared to those without land titles. One possible explanation for this result could be that land title provides youth with greater security and confidence in investing in their farms [51]. With formalized land tenure, they may be more likely to make long-term investments in their farms, such as purchasing equipment, improving soil fertility, or growing high value short-term or perennial crops which could result in higher productivity and income gain in agriculture. On the other hand, youth without formal land tenure may be hesitant to invest in their farms due to the risk of losing their land, which could result in less time being spent on farming activities.

**Table 2:** Descriptive results on youth characteristics by land titling status.

Variable	Non-titled	Titled	Total
Agric participation (hours per week)	17.597	21.728***	17.759
Non-farm business (% of youths in each group)	0.188	0.318***	0.194
Education level (years of formal education)	5.857	6.111***	5.869
Land holding size (acres)	3.535	3.419	3.530
Female-head (1 = yes, O=Otherwise)	0.241	0.246	0.242
Cooperative Member (1 = yes, O=Otherwise)	0.355	0.439***	0.359
Formal employment ((1 = yes, O=Otherwise)	0.003	0.008***	0.004
Plot-Market distance (Kilometers)	9.535	10.957***	9.598
Home-plot distance (Kilometers)	6.932	11.289***	7.123
SACCOS Member (1 = yes, O=Otherwise)	0.169	0.212***	0.171
Head's age (Years)	44.590	44.244	44.575
Youth_head (1 = yes, O=Otherwise)	0.149	0.087***	0.147
Other land docs (1 = yes, O=Otherwise)	0.169	0.725***	0.205
Dwelling title (1 = yes, O=Otherwise)	0.118	0.264***	0.125
HHSize_AE (Count)	4.846	5.363***	4.869

Note: Figures in parentheses are robust standard errors; the asterisks (*,** and ***) indicate a statistically significant difference at 10%, 5% and 1% respectively.

Kosec et al. [18] found the similar results is their study in rural Ethiopia arguing that youth who expect to gain land access and ownership rights are less likely to migrate from their localities and more likely to spend more of their time in agriculture than non-farm activities and the decisions appear to be driven by the expected gains from migration. Furthermore, Lambert et al. [52] point out, for the case of Senegal, that land ownership mitigates both the tendency to migrate and the tendency to diversify out of agriculture especially among youth who lack the responsibilities of being a head.

The results also show a significant difference in the proportion of youth business owners with land titles (0.318) compared to those without land titles (0.187). This could mean that youth with secure land tenure may have greater access to credit and other resources needed to start and sustain a non-farm business [15]. On the other hand, youth without land titles may have limited access to credit and resources, which could make it more difficult to start and sustain a non-farm business. Additionally, without secure land tenure, they may have fewer assets to use as collateral for loans or investments [53]. Nonetheless, there are varying perspectives around this aspect with others conflicting with our findings. For example, Kosec et al. [18] found relatively lower proportion of land certificate holders who own non-farm activities attributing that to higher security of tenure leading to more time spent on agricultural investments.

The descriptive results, further, suggest that youth who have land titles are more educated than those who do not have land titles. The data indicates that the average years of formal schooling for youth with titled land is 6.111 years, while those without titled land have an average of 5.857 years of formal schooling. There could be several possible explanations for this finding. One possible explanation is that land title ownership may serve as collateral for loans, enabling families to invest more in their children's education [54,55]. Another explanation is that land title ownership may provide a sense of stability and security, which could facilitate children's academic performance. Moreover, it's possible that those who have access to land title ownership may come from families with higher socio-economic status, which could also be linked to higher educational attainment. Msangi et al. [51] found that a relatively higher proportion of titled plots belong to more educated household heads than those without title, the main reason being that individuals with higher levels of education may be more not only capable of navigating the bureaucratic complexities of Tanzania's land titling systems but also are likely to have greater access to opportunities for remunerative formal wage employment.

The descriptive results presented in Table 2 reveal that there is no statistically significant difference in land holding size between youth with land titles and those without. On average, youth with titled land have a land holding size of 3.535 acres, while those without titled land have a slightly lower average of 3.418 acres. These findings suggest that land title ownership does not necessarily confer larger land holding advantage.

Moreover, the results indicate that there is no difference in the sex composition between youth with land titles and those without. Specifically, the proportion of female youth in each group is about 0.24, suggesting that gender does not appear to be a factor in land title ownership among youth. However, this is subject to confirmation from regression results.

### 3.2 Econometric results

This study run an IPWRA to investigate the extent to which land titling is influencing youth participation in Agriculture. The IPWRA helps to address potential endogeneity in the youth household landholding variable. Other statistical checks evolve around testing the model for multicollinearity and outliers as well as the assumption of linearity.

The preliminary data analysis was conducted which incorporated diagnostic tests to check for multicollinearity and validate the linearity assumptions. No serious issues were identified, affirming the reliability of the initial findings. Detailed results from these diagnostic tests are available upon request.

#### 3.2.1 Determinants of land titling decision among youth in Tanzania

This section presents the results of the logit selection equation, which explores the drivers of youth land titling decisions. The Logit results presented in Table 3 indicate a pseudo-R-squared value of 0.3835. This value implies that approximately 38.35% of the variation in the dependent variable is explained by the model. A higher pseudo-R-squared suggests a better fit of the model, implying that the included covariates collectively contribute significantly to explaining the variability in the youth number of hours youth spent in agriculture per week. Furthermore, our findings in Table 3 indicate several significant factors that influence youth decisions to obtain land titles. These results suggest that land titling decisions among youth are not random but are instead influenced by a systematic set of characteristics. If these characteristics are not accounted for, they may lead to selection biases. Specifically, our analysis shows that factors such as youth access to non-farm income, formal employment, education, and geographical location of the farm are among significant predictors of youth land titling decisions. These findings underscore the need to account for systematic factors that may influence land titling decisions among youth. A brief discussion of these results is presented below:

**Table 3:** Logit selection model results on the determinants of youth land titling decision in Tanzania.

Variables	Coefficient	Robust.Std.Err	Sig.
Dwelling_Cert	0.081	0.012	0.000
Govt_Employee	0.022	0.009	0.015
HHSize_AE	0.006	0.001	0.000
Marital_Widow_er	−0.127	0.002	0.000
_Head_Move_from_Other_Region	0.011	0.008	0.142
Cooperative	0.055	0.008	0.000
SACCOS	−0.003	0.011	0.761
Formal_Employment	0.919	0.025	0.000
Ln_Distance_Market_Plot	−0.024	0.004	0.000
Ln_Distance_Dwelling_Plot	−0.024	0.004	0.000
Ln_Plot_Year_Acquired	0.095	0.013	0.000
Female_Head	0.010	0.009	0.265
Other_Land_Certificates	0.182	0.009	0.000
Plot_Year_Aquired	−0.002	0.001	0.000
Ln_Farm_Size	0.014	0.003	0.000
Mechanization	0.072	0.014	0.000
Youth_Head	−0.097	0.042	0.019
Plot_Age	0.001	0.001	0.089
Head_Age	0.000	0.000	0.698
Ln_Montly_wage	0.000	0.001	0.617
_cons	−0.506	0.042	0.000
Number of obs.	5480		
Wald chi2 (17)	897.80		
Prob > chi2	0.000		
Pseudo R2	0.3835		
Log pseudolikelihood	−904.598		

Firstly, our results show that youth's possession of a dwelling land certificate is a significant determinant of their land titling decisions. Marginal effect coefficient is 0.081, and it is statistically significant at the 1% level. These findings suggest that youth who have already secured their dwelling land tenure are more likely to title their agricultural land plots due the possible exposure to similar procedures for agricultural land title acquisition. This finding is consistent with the notion that secure land tenure promotes investment in land and leads to increased productivity [51].

Furthermore, youth access to formal employment emerges as a pivotal factor influencing land titling decisions, highlighted by a substantial marginal effect coefficient of 0.919. This coefficient signifies that the availability of formal waged employment, whether through public or private sectors, equips households with essential income, skills, influence, and social networks. These assets are crucial for navigating the intricate and bureaucratic procedures for obtaining formal land titles within Tanzania's land administration systems [56]. A similar finding has been observed by Ref. [57] in the context of Ghana. However, the findings of this nature also underscore the urgent need for devising alternate strategies to promote youth land titling, particularly considering the limited accessibility of formal employment opportunities for numerous young individuals in Tanzania.

Another notable finding is that the size of the youth household significantly influences their land titling choices. The value for the marginal effect is 0.006, with a high level of statistical significance at the 1% level. This suggests that larger youth households are more inclined to prioritize obtaining secure land tenure, aiming to ensure their sustained well-being over time [51]. This trend could also be influenced by the cultural and societal significance attached to land ownership in many Sub-Saharan African countries, where possessing land is closely linked to social identity, economic stability, and community status [46,1].

Moreover, the study reveals that youth hailing from widow households exhibit a reduced likelihood of land titling, with a significant marginal effect of 0.127. This result potentially underscores the heightened vulnerability of widow households and their limited access to resources in many SSA countries [58,59]. The significance of this finding points to the need for re-designing and effective implementation of gender-sensitive policies and targeted land titling initiatives, aiming to bolster the impact on vulnerable youth segments, particularly those within widow households [56]. This approach can play a pivotal role in addressing inequalities in land tenure and promoting equitable land tenure rights.

Adding to the findings, the study reveals that cooperative membership bears a substantial positive influence on youth land titling decisions, as depicted by the marginal effect of 0.055. This observation suggests a crucial role played by cooperatives in propelling land titling among youth, potentially facilitated by augmented information access, collective endeavors, and resource accessibility, including credit facilities, shared among cooperative members [60]. This implication further highlights the potential positive spillover effects on youth land tenure resulting from government initiatives aimed at fostering cooperative development [56]. Cooperative membership evidently contributes to enhancing the overall landscape of youth land ownership.

Moving forward, our analysis as displayed in Table 3 unveils a noteworthy observation: a negative impact (with marginal effect value of −0.024) linked to the distance between the nearest market center and farm plots on youth land titling choices. This implies that youth are potentially unwilling to pursue land titling for plots situated farther away from market centers due to low competitiveness and potential for agricultural investments associated with plots distant from market hubs [51]. This is largely due to the inherent expense of accessing factor and product markets, leading to escalated production costs [56]. Alternatively, this phenomenon can be explained by the challenging access to credit in such remote areas where financial institutions face difficulties in monitoring credit transactions in such areas, resulting in limited availability of investment capital for land plots in these regions [31]. Consequently, this finding underscores the critical importance of infrastructure development and well-considered land use planning, particularly in relatively more remote areas. These strategies are essential to catalyze land titling efforts, promote agricultural investment, and alleviate the barriers posed by geographical isolation [61,62].

Conversely, the distance between the youth's dwelling and the plot bears a significantly negative effect (with marginal effect of −0.024) on youth land titling choices. This finding suggests that land plots situated far from youth's residence are more likely to lack secure tenure. In such cases, alternative land protection mechanisms, such as those explored by Hombrados et al. [63], might prove ineffective. As a result, land titling seems to be the primary and possibly sole viable option. Furthermore, the negative coefficient highlights the notion that securing formal land titles becomes especially imperative to youth when the proximity to home cannot be relied upon as a safeguard against potential land disputes or encroachments.

Our logit model results, further, indicate that the number of years since the land plot was acquired has a significant effect on youth land titling decisions in Tanzania, with a marginal effect of −0.002. The negative coefficient value suggests that as the number of years since acquisition of the land plot increases, the probability of titling it decreases for youth. This implies that younger generations are more likely to prioritize formalizing their land tenure through titling on plots acquired recently at a high risk of insecurity, while older plots probably inherited from parents where such risk is less obvious [51].

The study, also, finds a significant and positive effect of youth land holding size and their decisions regarding land titling (with marginal effect of 0.014). This interesting pattern can be explained by considering the recently documented pattern of growing number of medium scale farmers (with farm size between 5 and 100 ha) in Sub-Saharan African agriculture, significant share of which are youth [64,65,66]. Although, agriculture is still taken as an alternative to unemployment in most SSA countries, many youths are increasingly treating farming as a business venture rather than just a subsistence activity [65]. This growing shift towards entrepreneurial farming implies a greater need for access to financing and credit facilities to support investments in equipment, technology, and market expansion [67]. In this context, formal land ownership assumes paramount importance. Youth with larger farm holdings may seek to leverage their land as collateral when approaching financial institutions for loans [51]. Formal ownership serves as a tangible and recognized asset that can be used to secure loans, indicating a higher level of commitment to agricultural development.

#### 3.2.2 Effect of land titling on youth participation in agriculture

In this section we present the IPWRA results on the effect of land titling on youth participation in agriculture defined by the number of hours per week in farming as adopted from IFAD definition in Kafle, Paliwal and Benfica [65]. The findings presented in Table 4 suggest that land titling is an important factor that affects youth participation in agriculture in Tanzania. The reported ATT estimates show a significant positive effect of land titling on the amount of time that young people devote to farming activities. This means that young people who have their land titled tend to allocate more time to farming activities than those who do not. The estimated ATT value of 6.069 h per week implies that land titling decisions among youth increase the amount of time they allocate to farming by this amount on average. This finding confirms the results from the descriptive statistics and it has important implications for policy and programs such the currently running program by the Government of Tanzania for building an entrepreneurial mindset through agri-business technical and vocational training, the Building Better Tomorrow (BBT) [68], and other interventions aimed at promoting youth participation in agriculture, as increasing access to land through land titling programs can help increase agricultural productivity and contribute to food security and household income in Tanzania. The findings that young people's allocation of time between farming and off-farm activities is significantly influenced by land titling is consistent with previous studies [69,18] and is therefore not surprising.

**Table 4:** IPWRA results on the effect of land titling on youth participation in Agriculture in Tanzania.

Variables	Titled			Non-titled		
	Coefficient	Robust.Std.Err	Sig.	Coefficient	Robust.Std.Err	Sig.
Educ_Above form 4	20.281	2.924	0.000	−10.899	2.924	0.000
Educ_Above primary	0.823	2.067	0.690	−5.411	2.067	0.690
Youth hhead	18.263	1.991	0.000	−10.455	1.991	0.000
Land dispute	−0.557	1.653	0.736	−40.171	1.653	0.736
Plot owner's age	−0.995	0.130	0.000	1.639	0.130	0.000
HHead's age	−0.550	0.059	0.000	−0.159	0.059	0.000
Urban resident	−17.822	1.088	0.000	−0.201	1.088	0.000
Ln_Year plot acquired	4.519	0.864	0.000	−7.118	0.864	0.000
Cooperative	5.905	0.974	0.000	2.845	0.974	0.000
SACCOS	9.428	1.257	0.000	−30.289	1.257	0.000
Ln_Distance_home_plot	−5.425	0.694	0.000	−12.673	0.694	0.000
Ln_Distance_market_plot	−8.323	0.876	0.000	−13.992	0.876	0.000
Female_head	14.794	1.213	0.000	15.835	1.213	0.000
Marital_married	−2.458	0.283	0.000	−0.562	0.283	0.000
Non-farm business	1.958	0.883	0.027	−18.011	0.883	0.027
Farm_Size	8.746	2.211	0.385	6.022	4.601	1.541
Mechanization	−0.450	1.387	0.746	11.300	1.387	0.746
HHSize_AE	1.817	0.139	0.000	3.211	0.139	0.000
Ln_Montly_wage	−0.573	0.130	0.000	−0.146	0.130	0.000
_cons	39.062	5.317	0.000	1.867	5.317	0.000
**ATT**	**6.069**	**1.396**	**0.000**			
**POmean**	**19.491**	**1.215**	**0.000**			

Note: POmean is the potential outcome mean number of hours per week spent in agriculture for non-treated youth farmers.

We also present the results of the covariates of land titling on youth participation in agriculture.

Our findings show that the size of the farm being cultivated is a significant factor affecting youth involvement in agriculture in Tanzania. The coefficient estimates of land size (8.746) indicates that increasing the acreage of farmland by one can boost youth participation in agriculture by an average of 8.746 h. These findings shed light on how land ownership concentration could impact the amount of time dedicated to farming by youth, which has important implications for initiatives likes BBT that aims to train and assign 10 or more acres of agricultural land and other resources to young people as a means of encouraging their participation in agriculture. The positive effects of land ownership concentration suggest that pro-youth programs like BBT have great potential to promote youth involvement in agriculture, which can fuel growth and transformation in the sector.

Educational level emerges as another key determinant. Higher formal education reduces weekly farming hours. Impact heightens with education levels, e.g., completing primary education reduces hours by 5.411, and secondary education by 10.899 (Table 4). Formal education equips youth with foundational skills crucial for formal employment. Higher education enhances off-farm prospects and uplifts aspirations, notably in societies where farming's social status is lower [70]. In many developing regions, education's seen as a pathway beyond agriculture. This holds true even where off-farm jobs are scarce. Youth with advanced formal education often favor non-agricultural employment [71]. These factors intricately shape the education-youth-agriculture relationship.

Land dispute experience significantly impacts youth participation in agriculture. The coefficient reflects that having encountered land disputes notably reduces youth's agricultural engagement. This finding holds vital implications for agricultural progress, especially in regions prone to such disputes. The observed effect's magnitude is substantial; youth with dispute experience spend 40.171 fewer hours farming weekly. This suggests disputes profoundly influence their decision to participate. One possible reason could be that disputes foster uncertainty and insecurity among youth in agriculture. This uncertainty impedes long-term investment, dampening their motivation, thus, reduced farming time becomes apparent [51]. This point to the need for policies and strategies addressing land disputes, with particular attention to areas prone to land disputes. Viable strategies encompass offering legal aid to affected youth or preemptive measures to avert disputes.

Our analysis reveals an interesting finding that being an urban resident has a significant negative effect on youth participation in agriculture, but this effect is contingent on land ownership status. Specifically, the negative impact of urban residence on youth involvement in agriculture is statistically significant for those who do not hold formal land titles, with an average reduction of 17.822 h per week. However, for those who own land titles, the effect is not statistically significant, with a negligible reduction of 0.201 h per week on average. The findings of this nature could imply that urbanization may lead to a shift in youth aspirations and preferences towards non-agricultural activities, such as education or formal employment, especially among those who do not own formal land titles. Lack of land ownership may limit access to credit, inputs, and markets, which are essential for successful farming. Thus, youth who do not have formal land titles may find it challenging to continue farming while living in urban areas, which could explain the significant reduction in their participation in agriculture.

The study reveals that the distance separating the dwelling and farm plot has a significant impact on the involvement of youth in agriculture in Tanzania. According to the coefficient estimate, an increase in the distance from home to the farm plot by 1-km results in an average reduction of 12.672 h per week in the time youth spend on farming activities. This finding is reasonable since a long distance can impose practical challenges that hinder youth from participating in agriculture. For instance, traveling back and forth may consume a lot of time and energy, causing fatigue and discouraging them from pursuing farming. Moreover, high transportation costs may lower the profitability of agricultural activities, making it less appealing to youth. Additionally, the distance between the dwelling and farm plot may affect the youth's ability to manage and supervise farming activities. If the farm plot is far, it becomes harder to monitor crops, manage pests, and perform other necessary tasks, resulting in low yields, reduced profits, and diminished motivation to continue with agriculture. Similar results were reported in a study conducted in Uganda which reveals that the distance from the household to the farm plot was a significant factor affecting youth participation in agriculture. Specifically, the study found that a 1 km increase in the distance to the farm reduced the likelihood of youth engaging in agriculture by 16.2% [72]. Another study in Ethiopia found that the distance between the household and the farm plot was negatively correlated with the participation of youth in agricultural activities. The study found that youth living farther from the farm plots tended to spend less time on agriculture [73].

We further present in Table 4 that the distance between the farm plot and the nearest market is a crucial determinant of youth participation in agriculture in Tanzania. The results show that 1 km increase in farm-to-market distance decreases the average time youth spend on agricultural activities by 8.323 h and 13.991 h per week for titled and non-titled youth respectively. This implies that access to markets has a positive impact on youth participation in agriculture. One possible explanation for this result is that youth farmers who have shorter distances to markets have more opportunities to sell their produce and generate income. This, in turn, could motivate them to continue participating in agricultural activities [20,71]. Moreover, short the farm-to-market distance reduces transportation costs, which may increase the profitability of farming activities and attract more youth to engage in agriculture. Another possible explanation is that a shorter distance to the market may allow youth farmers to access agricultural inputs and services more conveniently. This, in turn, can improve their productivity and lead to higher yields and profits.

The study suggests that youth from female-headed households in Tanzania tend to spend significantly more time in agricultural activities compared to youth from male-headed households. Specifically, the results indicate that youth from female-headed households spend 15.834 h per week more on average in agriculture when they have a land title, and 14.794 h per week more on average when they do not have a land title. The result of this nature could be explained by the fact that female-headed households face greater economic challenges and have fewer opportunities compared to male-headed households, leading youth to engage more in agriculture as a means of supporting their family's livelihoods. Additionally, female-headed households may have limited access to other sources of income, such as formal employment, which further reinforces the need to rely on agriculture as a primary source of income. Furthermore, female-headed households may face gender-specific constraints that limit their access to other forms of income and economic opportunities. For example, they may have limited access to credit, which can affect their ability to invest in agriculture and increase productivity. In some cases, they may also face discrimination and limited access to land and other resources, which can further constrain their economic opportunities and increase their reliance on agriculture. Overall, the study highlights the importance of gender-sensitive policies and interventions that aim to improve the economic opportunities of female-headed households in Tanzania. By addressing gender-specific constraints and improving access to resources and opportunities, it may be possible to reduce the economic challenges faced by female-headed households and promote more sustainable and equitable participation of youth in agriculture.

The results suggest that access to non-farm income has contrasting effects on the amount of time youth spend in agriculture, depending on whether they have land titles or not. For youth without land titles, access to non-farm income significantly reduces the amount of time spent in farming, by an average of 18.011 h per week. However, for youth with land titles, access to non-farm income increases the amount of time spent in agriculture, by an average of 1.958 h per week.

The likely explanation for these contrasting results is that youth with land titles may be more confident in their land tenure security, and therefore more willing to invest in non-farm activities that could provide an alternative source of income [51]. By contrast, youth without land titles may feel more vulnerable and insecure about their access to land, and thus may be more likely to focus on farming activities as a means of securing their livelihoods. Access to non-farm income may not be seen as a viable alternative for them, especially if they lack the collateral needed to obtain credit or financing for non-farm enterprises. An alternative but complementary explanation is that youth with land titles may have greater access to information, networks, and resources that enable them to pursue non-farm opportunities [66]. For example, they may have greater access to markets, transportation, and information about business opportunities. Youth without land titles, on the other hand, may face greater barriers to accessing information and resources, which may limit their ability to pursue non-farm income-generating activities [71]. Generally, the results suggest that land tenure security plays an important role in shaping youth labor patterns and their ability to access non-farm income.

## 4 Conclusion, recommendations and areas for further research

### 4.1 Conclusion

In conclusion, the findings presented in this study suggest that land titling is an essential factor in influencing youth participation in agriculture in Tanzania. The study found that land titling has a significant positive effect on the amount of time that young people allocate to farming activities. This indicates that young people who have their land titled tend to spend more time on farming activities than those who do not have land titles. Additionally, the size of the farm being cultivated, the educational level of youth, and land dispute experience are crucial factors affecting youth participation in agriculture. The study recommends that policies and programs aimed at promoting youth participation in agriculture should consider increasing access to land through land titling programs, addressing land disputes, and promoting formal education. Furthermore, pro-youth programs like BBT that aim to assign 10 or more acres of agricultural land to young people have great potential to promote youth involvement in agriculture, which can contribute to growth and transformation in the sector.

On the methodological side, the results from the first stage analysis using the Logit model suggest that land titling decisions among youth are not random but are instead influenced by a systematic set of characteristics. If these characteristics are not accounted for, they may lead to selection biases. Therefore, the second stage analysis using the doubly robust IPWRA approach to tackle the aforementioned estimation issue is critical in this type of analysis yet often overlooked in the body of literature.

### 4.2 Recommendations

Based on the findings presented in this study, we recommend the following interventions to promote youth participation in agriculture with potential impact on agricultural development in Tanzania:

1. Land titling programs are an effective way to increase youth participation in agriculture. Therefore, the government and other stakeholders should invest in land titling programs that specifically target young people. Such programs can increase access to land, which can provide young people with a source of income and contribute to food security and rural development.
2. Pro-youth programs such as the currently running BBT, have great potential to promote youth participation in agriculture in Tanzania. Therefore, we recommend that relevant stakeholders including the Government agencies, agricultural cooperative societies, academic institutions, non-governmental organizations and financial institutions increase their investment in pro-youth programs, especially those that provide training and resources to young people to help them succeed in agriculture.
3. Education: Education is a critical factor in determining youth participation in agriculture. Education programs that equip young people with foundational skills such as cognitive, numeracy, and literacy skills.
4. Land disputes have a significant negative impact on youth participation in agriculture. Policies and programs are needed that address land disputes in agricultural communities such as legal assistance and support to youth who have experienced land disputes or implement measures to prevent such disputes from occurring in the first place.
5. Tailored interventions are recommended for young people who do not have formal land titles: Our analysis reveals that the effect of urban residence on youth participation in agriculture is contingent on land ownership status. Therefore, interventions that focus on increasing access to credit, inputs, and markets would help them succeed in agriculture.

Overall, the recommendations provided above are crucial for promoting youth participation in agriculture in Tanzania. Programs with increasing access to land, providing training and resources, promoting education, addressing land disputes, and tailoring interventions can support young people to succeed in agriculture, which ultimately contribute to food security, job creation, rural development, and economic growth of the nations.

### 4.3 Areas for further research

While this study offers important contribution into the nexus between land titling and youth participation in agriculture in Tanzania, several avenues for further research could broaden our understanding and enhance policy recommendations. In that regard, the following areas are suggested for further research:

1. *Long-Term Impact Assessment:* Conducting longitudinal studies to assess the sustained impact of land titling on youth participation in agriculture over an extended period would provide a more in-depth understanding of the dynamic nature of this nexus. This could involve tracking the trajectories of youth participation in land titling programs over several years to capture the evolving patterns and outcomes.
2. *Qualitative Exploration of Motivations:* Supplementing quantitative findings with qualitative research methods, such as in-depth interviews or focus group discussions, can unveil a more useful insights into the motivations behind youth participation in agriculture in Tanzania and other relevant contexts. In-depth understanding of the underlying factors that drive or hinder youth involvement in agriculture can inform targeted interventions and relevant policy adjustments.
3. *Regional Variation Analysis:* Investigating regional variations in the impact of land titling on youth participation could reveal context-specific dynamics. Different agro-ecological zones or varying levels of urbanization in the country may potentially influence the effectiveness of land titling programs, necessitating more tailored strategies for economically and economically optimal outcomes.
4. *Gender Dimensions:* Examining the gender-specific effects of land titling on youth engagement in agriculture would contribute to a more inclusive understanding. Research could explore whether the impact varies for young men and women, considering the potential differential access to resources and opportunities.
5. *Exploration of Potential Policy Synergies:* While this study has shown a positive effect of land titling on youth participation in agriculture, the impact of land titling interventions may not be fully realized in absence of other potentially complementary interventions. Investigating potential synergies between land titling and different policy interventions such as combining land titling programs with education initiatives, dispute resolution mechanisms, input subsidies and/or social protection interventions, could not only provide a more robust evidence-based policymaking to promoting youth participation in agriculture but also contribute to the ongoing dialogue on promoting sustainable agricultural development and youth empowerment in Tanzania and other Sub-Saharan African countries.

## Ethics declaration

Review and/or approval by an ethics committee was not needed for this article does not contain any studies with human participants performed by any of the authors. Informed consent was not required for this study because this article does not contain any studies with human participants performed by any of the authors.

## Funding disclosure

This study was funded by International Institute of Tropical Agriculture (IITA) under the project “Youth Researching Youth: Competitive Fellowships for Young African Scholars Researching Youth Engagement in Rural Economic Activities in Africa” from a Grant number 2000001374 by the International Fund for Agricultural Development (IFAD).

## Data availability statement

Most of the datasets utilized in this study are publicly accessible in a secure version via this link: https://microdata.worldbank.org/index.php/catalog/2862. However, for a few variables not present in the secured version, an unsecured version is used but it is not publicly available due to data protection policies of the Tanzania National Bureau of Statistics and the World Bank. Nonetheless, the unsecured data version can be provided upon reasonable request from the respective organizational authorities.

## CRediT authorship contribution statement

**Haji Athumani Msangi:** Writing – original draft, Visualization, Validation, Methodology, Investigation, Funding acquisition, Formal analysis, Data curation, Conceptualization. **Betty Waized:** Writing – review & editing, Visualization, Validation, Supervision, Methodology, Conceptualization. **Daniel Wilson Ndyetabula:** Writing – review & editing, Visualization, Validation, Supervision, Methodology, Investigation, Conceptualization. **Victor M. Manyong:** Writing – review & editing, Writing – original draft, Visualization, Validation, Supervision, Software, Resources, Project administration, Methodology, Investigation, Funding acquisition, Conceptualization.

## Declaration of competing interest

Haji Athumani Msangi reports financial support, administrative support, article publishing charges, and travel were provided by International Fund for Agricultural Development. Haji Athumani Msangi reports financial support, administrative support, statistical analysis, travel, and writing assistance were provided by International Institute for tropical Agriculture. If there are other authors, they declare that they have no known competing financial interests or personal relationships that could have appeared to influence the work reported in this paper.
